# Orthogonal Thin Film Photovoltaics on Vertical Nanostructures

**DOI:** 10.1186/s11671-015-1187-6

**Published:** 2015-12-16

**Authors:** Arman Ahnood, H. Zhou, Y. Suzuki, R. Sliz, T. Fabritius, Arokia Nathan, G. A. J. Amaratunga

**Affiliations:** School of Physics, University of Melbourne, Melbourne, Australia; Peking University Shenzhen Graduate School, Peking University, Shenzhen, China; London Centre for Nanotechnology, University College London, London, UK; Optoelectronics and Measurement Techniques Laboratory, University of Oulu, Oulu, Finland; Electrical Engineering Division, Department of Engineering, University of Cambridge, Cambridge, UK

**Keywords:** Thin film solar cells, Orthogonal solar cells, Illumination uniformity, Series resistance, Electric field confinement

## Abstract

Decoupling paths of carrier collection and illumination within photovoltaic devices is one promising approach for improving their efficiency by simultaneously increasing light absorption and carrier collection efficiency. Orthogonal photovoltaic devices are core-shell type structures consisting of thin film photovoltaic stack on vertical nanopillar scaffolds. These types of devices allow charge collection to take place in the radial direction, perpendicular to the path of light in the vertical direction. This approach addresses the inherently high recombination rate of disordered thin films, by allowing semiconductor films with minimal thicknesses to be used in photovoltaic devices, without performance degradation associated with incomplete light absorption. This work considers effects which influence the performance of orthogonal photovoltaic devices. Illumination non-uniformity as light travels across the depth of the pillars, electric field enhancement due to the nanoscale size and shape of the pillars, and series resistance due to the additional surface structure created through the use of pillars are considered. All of these effects influence the operation of orthogonal solar cells and should be considered in the design of vertically nanostructured orthogonal photovoltaics.

## Background

**Fig. 1:**
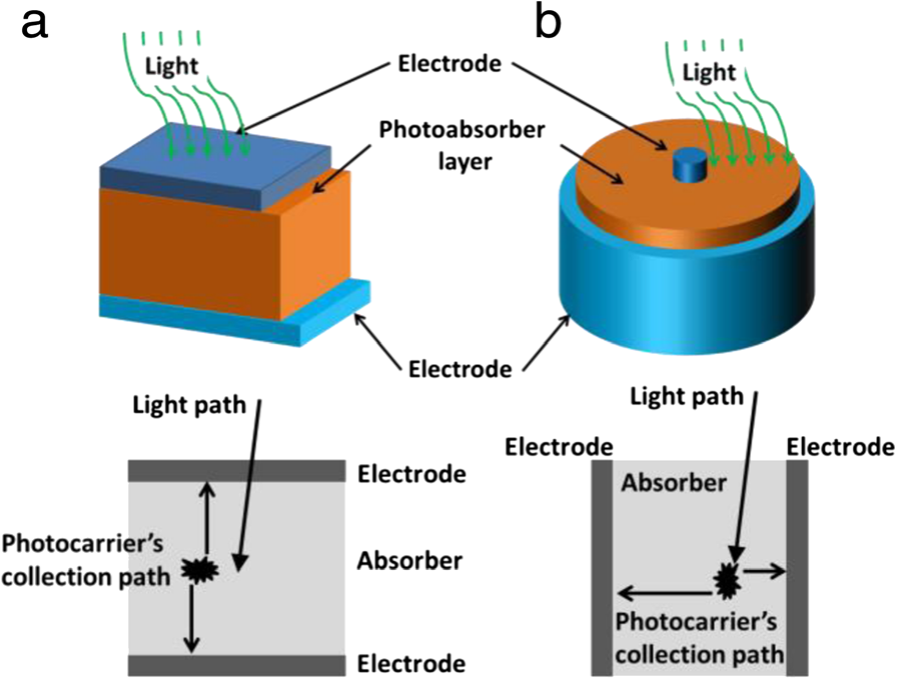
Cross-sectional diagram of **a** planar solar cell and **b** orthogonal solar cell. As illustrated, in the case of planar solar cells, photocarrier collection path is in the same direction as the light path. However, orthogonal solar cells allow the photocarrier’s collection path to be decoupled, in this case perpendicularly, from the optical path. This makes it possible to use a thin photoabsorber layer, for enhance the collection efficiency, while maintaining the necessary length of optical path to prevent losses associated with incomplete light absorption

Thin film photovoltaic devices, also known as the second generation solar cells, have provided a complimentary platform to the first generation solar cells based on bulk materials, by catering for the low cost, and low efficiency applications [1]. Orthogonal solar cells, a subgroup of the third generation solar cells, are an extension of the thin film solar cells and operate based on the principle of perpendicular path of illumination with respect to photocarrier collection path [2, 3]. In conventional thin film photovoltaic devices, light travels in the same direction as the photogenerated carriers within the absorber layer as illustrated in Fig. 1a. Here, photogenerated carrier lifetime imposes a design limit on the upper value of the absorber layer thickness. This typically leads to incomplete light absorption, as maximizing the light absorption requires increasing the thickness of the absorber layer. Conventional thin film solar cells’ photoabosorber layer thickness is optimized to minimize the recombination losses while maximizing the light absorption [4]. In addition to this, optical enhancements such as textured electrodes, back reflectors, and anti-reflective coatings serve to further improve the light absorption without increasing absorber layer thickness and subsequently prevent the increase in recombination of the photogenerated carrier [5]. Solar cells with an orthogonal structure offer an alternative solution to address this challenge. The structure of such device is shown in the Fig. 1b. It consists of thin film photovoltaic devices grown on an array of vertically aligned nanopillars [6, 7] and other vertical nanostructures such as spikes [8, 9]. Here, decoupling of photogenerated carriers and optical light pathways allows the use of a thin absorber layer to maximize the collection of the photogenerated carriers, while providing sufficient depth for complete light absorption [10, 11].

Despite the simplicity of the concept of orthogonal solar cells, there are a number of underlying physical mechanisms which need to be accounted when considering the form factor of orthogonal solar cells. These require development of a design framework which is tailored for the orthogonal devices based on the physical effects uniquely present in this class of devices. Earlier works have demonstrated the clear influence of pillar height and diameter on the efficiency of thin film orthogonal solar cells [3, 12]. This paper builds on the earlier works by considering (i) non-uniformity of the illumination across the depth of the device, (ii) electric field enhancement effects at the nanoscales, and (iii) increased series resistance due to the higher device surface area.

## Methods

The test structures were fabricated in this study consisted of silicon thin film PV cells deposited on vertical nanostructures and on a flat ITO-coated glass substrate as reference samples. Where vertical nanostructures were used, they consisted of either an array of MWCNTs or ZnO nanowires with their growth deposition methods reported in earlier works [6, 13]. The PV cells consisted of p-i-n type structure deposited using plasma-enhanced chemical vapor deposition with their deposition methods reported in earlier works [14]. The thicknesses of active layers used here are p-type amorphous silicon carbide (20 nm), intrinsic a-Si:H (300 nm), n-type nanocrystalline silicon (40 nm). PV cell measurements were performed using Keithley source meter 2400, under dark and various illuminated conditions. Simulations were performed using SPICE based module on a double diode circuit module with series and parallel parasitic resistances (AimSpice software).

## Results and Discussions

### Illumination Uniformity

**Fig. 2:**
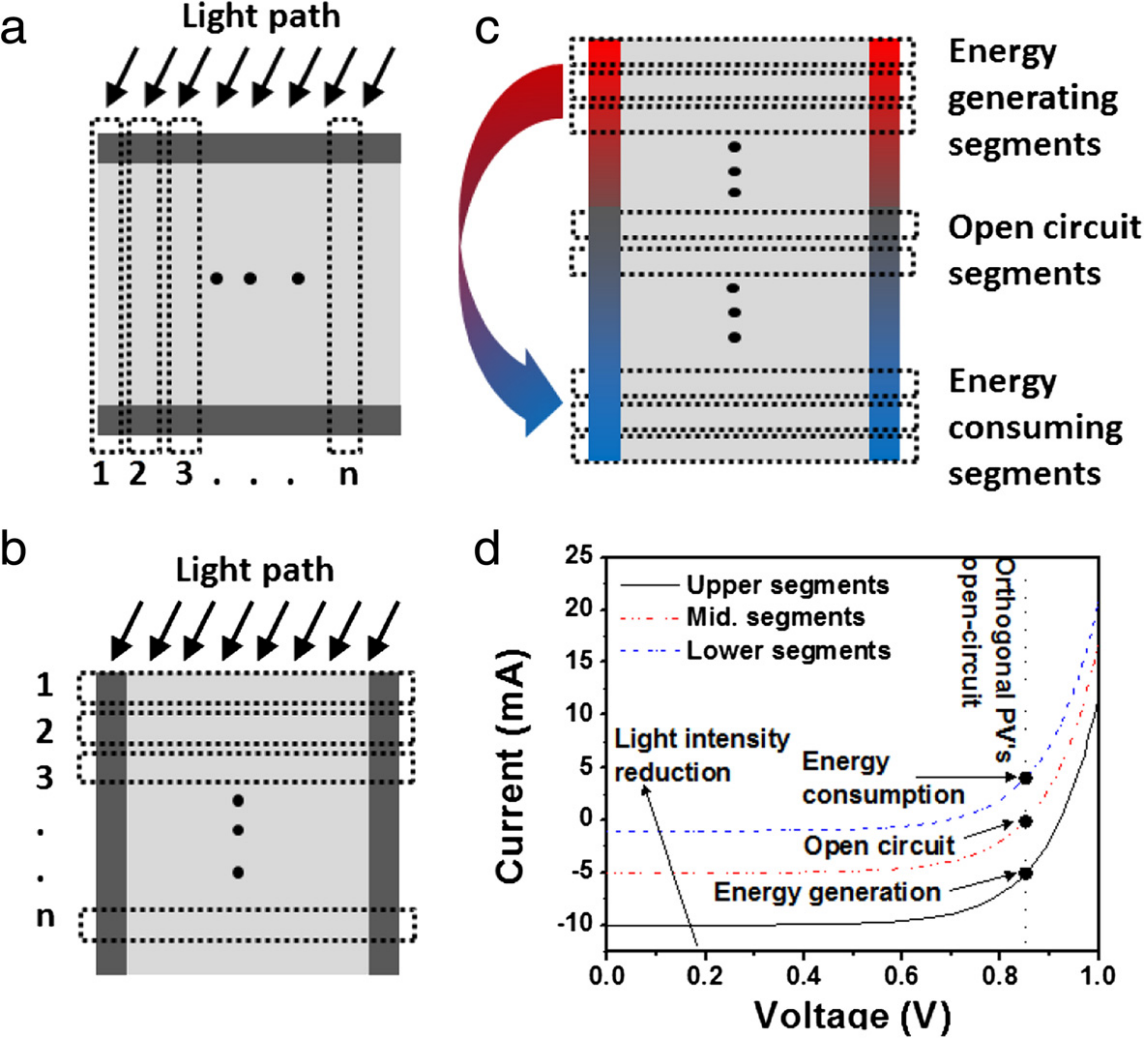
**a** Planar and **b** orthogonal cells can be modeled as an array of parallel connected solar cell segments. As illustrated, planar solar cell segments experience uniform illumination throughout the device. However, in the case of orthogonal solar cells, each segment acts as an optical filter and removes a portion of the light entering in the next segment. This leads to a non-uniform illumination intensity or spectrum at each of the orthogonal solar cell segments and analogy comparable to tandem solar cells. The consequence of this non-uniformity is illustrated in **c**, where orthogonal solar cell under illumination and operated at its maximum power point would comprise of energy generating segments, open circuit segments, and energy consuming segments. Lower segments are exposed to lower light intensity but operate at the same voltage as the upper segments as all the segments are parallel connected. The lower light intensity at lower segments and there operation voltage result in their operation in the energy consuming mode. The lower segments draw current and consume energy generated by upper segments in order to achieve the same output voltage. This is due to the variation in the current-voltage characteristics of different segments exposed to a variable illumination as highlighted in **d**

Conventional planar solar cells are two terminal electrical devices which can be considered as an array of parallel-connected smaller planar segments, as shown in Fig. 2a. In conventional solar cells, the segments are illuminated uniformity across the planar device, leading to uniform electrical characteristics for all segments. Assuming a spatially uniform illumination, the parallel configuration between each segment implies the open circuit voltage (V_OC_) and the fill factor (FF) of the solar cell is equal to that of the individual segments, while the short circuit current (I_SC_) is equal to the sum of all segment’s I_SC_.

Figure 2b shows the similar parallel-configured solar cell segments in the case of the orthogonal solar cells. As the light travels through the orthogonal device, a proportion of the illumination is absorbed in each segment, with the material absorption coefficient determining the proportion of light transmitted through to the next segment. This gives rise to variable illumination intensity at each segment of the orthogonal solar cell. Furthermore, the wavelength dependence of the absorption coefficient in many materials leads to a variation in the illumination spectrum reaching each segment. These differences in the illumination intensity and spectrum give rise to a variability in the electrical characteristics from the various segments of the orthogonal solar cell.

The first segment, closest to the point of light entering the orthogonal solar cell, experiences the highest intensity of illumination and therefore the highest V_OC_ and I_SC_ compared with the rest of the segments. Here, the illumination spectrum will be typically 1.5 AM spectrum, with the values of V_OC_, FF, and J_SC_ of the segment comparable to a planar solar cell with similar thickness. On the other hand, the final segment is exposed to a lower light intensity, thus exhibiting a lower V_OC_ and I_SC_ with a different FF compared with the first segments [15]. Although the variation in the electrical performance between different segments is marginal in short pillars, this variation is exacerbated in longer structures. As shown in Fig. 2c, this variability has a detrimental impact on the overall V_OC_ of orthogonal solar cell. All segments are connected in parallel, which infers the same V_OC_ for across all segments. The comparatively poor electrical performance of the lower segments, due to the weak illumination, leads to a reduction in the overall V_OC_ of the orthogonal device. An orthogonal solar device biased at the open circuit point will only have a small portion of its segments operating at the open circuit condition. The upper segments with a larger V_OC_ will still be generating power, which is then internally consumed by the solar cell itself in the lower segments with lower V_OC_, as depicted in Fig. 2c, d.

The relationship between the open circuit voltage of a given segment and the number of the preceding segments can be derived as following [16]. The current-voltage characteristics of solar cells can be approximated as Eq. 1. Here, *J* denotes the output current of the solar cell, *J*_0_ the diode reverse bias saturation current, *J*_*L*_ the current generated by the incident light, *V* the terminal voltage of the cell, $\frac{q}{kT}$ is ~1/25.6 mV at room temperature, and *A* is the diode’s ideality factor.

1 $$ J={J}_0\left[{e}^{\frac{qV}{AkT}}-1\right]-{J}_L $$

*J*_*L*_ is typically proportional to the light absorbed in the segment, which can be related to the segment thickness, *δL*, absorption coefficient, *α*, and the intensity of incident light, *I*_0_, as shown in Eq. 2.

2 $$ J={J}_0\left[{e}^{\frac{qV}{AkT}}-1\right]-{I}_0{e}^{-\alpha \delta L} $$

At the open circuit condition, *J* = 0 and thus Eq. 2. can be rearranged to Eq. 3.

3 $$ {V}_{{\mathrm{OC}}_n}=\frac{AkT}{q} \ln \left[\frac{I_{0_n}{e}^{-\alpha \delta L}}{J_0}+1\right] $$

where *I*_0*n*_ is the light intensity arriving at the *n*th segment which can be related to the light intensity entering the orthogonal solar cell device as *I*_0*n*_ = *I*_0_.*e*^(-*αnδL*)^. Given that the absorption coefficient *α* is material-depended parameter, deviation in the V_OC_ of different segments will depend to the type of the orthogonal solar cells. For instance in disordered thin film semiconductors, such as amorphous silicon, the *α* can be related to the wavelength based on the Tauc equation [17] as shown in Eq. 4. Here, *E*_*g*_ is the bandgap of the material, *hv* is the photon energy of the incident light in eV, *α* is the absorption coefficient of the material at the given wavelength and *C* is a constant,

4 $$ ahv=C{\left(hv-{E}_g\right)}^r $$

with *r* being 2 for the thin film silicon photoabsorber layer. This allows the calculation of the open circuit voltage as a function of wavelength for different segments of the orthogonal solar cell as shown in Eq. 5.

5 $$ {V}_{{\mathrm{OC}}_n}=\frac{AkT}{q} \ln \left[\frac{I_0{e}^{--\frac{C{\left(hv-{E}_g\right)}^2}{hv}\left(n+1\right)*\delta L}}{J_0}+1\right] $$

Equation 5 points to the dependence of the V_OC_ to the number of segments and therefore the length of the orthogonal solar cell as well as the illumination spectrum. This is to say that certain orthogonal solar cells are better situated in certain illumination spectrum.

**Fig. 3:**
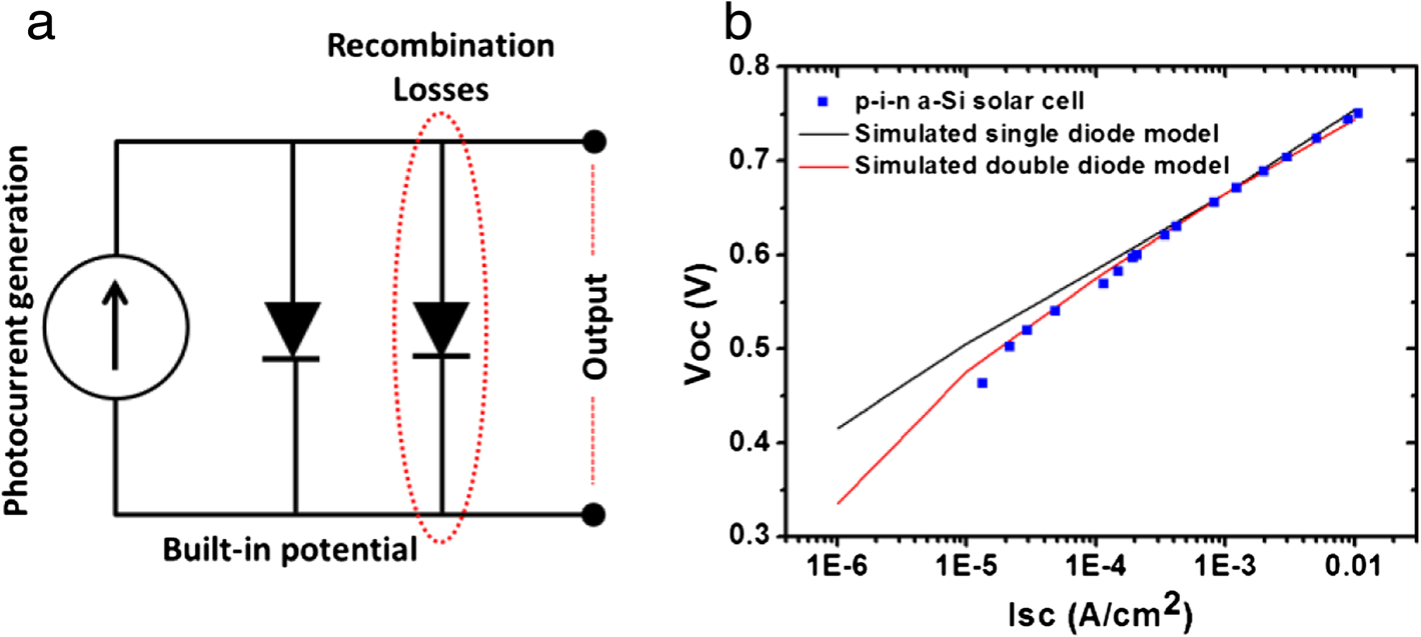
**a** Double diode equivalent circuit model is selected in this work to allow a more realistic variation in the solar cell characteristics with change in the illumination. A key variability in here is the change in the I_SC_ and V_OC_ with illumination intensity. **b** Illustrates dependence of the V_OC_ to I_SC_ in a p-i-n type planar a-Si:H thin film solar cell. The experiment points are attained with the variation in the light intensity over three order of magnitude. Simulation using double diode model can better account for the recombination losses which are dominant at lower light intensities compared to single diode model

In addition to the V_OC_, the I_SC_ and FF are also affected by the non-uniform illumination across the various segments of the orthogonal solar cell. Furthermore, Eq. 5 is based on a single diode model which cannot adequately describe certain classes of devices [18] particularly to illumination dependence of *J*_0_ [19]. These effects can be simulated by representing each of the segments using an electrical circuit model as shown in Fig. 3a. In here, a double diode model is used that allows a more realistic account for the carrier recombination in the absorber layer, which strongly influences device characteristics under low illumination intensity [20], particularly prevalent in the case of thin film materials [21]. The effect of variable illumination in each segment is accounted by varying the current source, *I*_*L*_ for each of the segments. The relative value of *I*_*L*_ is calculated based on the light absorbed in the semiconducting layer of each segment based on the Tauc equation assuming adequate spacing between the nanopillars to avoid optical scattering [22]. The simulation parameters were fitted to a fabricated p-i-n a-Si:H solar cell report earlier [14]. Figure 3b depicts the dependence of the V_OC_ to I_SC_, which is varied by changing the light intensity. The full orthogonal solar cell was modeled by connecting 100 segments with variable *I*_*L*_ in parallel. The 100-element approximation was verified by repeating the simulation with 1000 segments which yielded the same results.

**Fig. 4:**
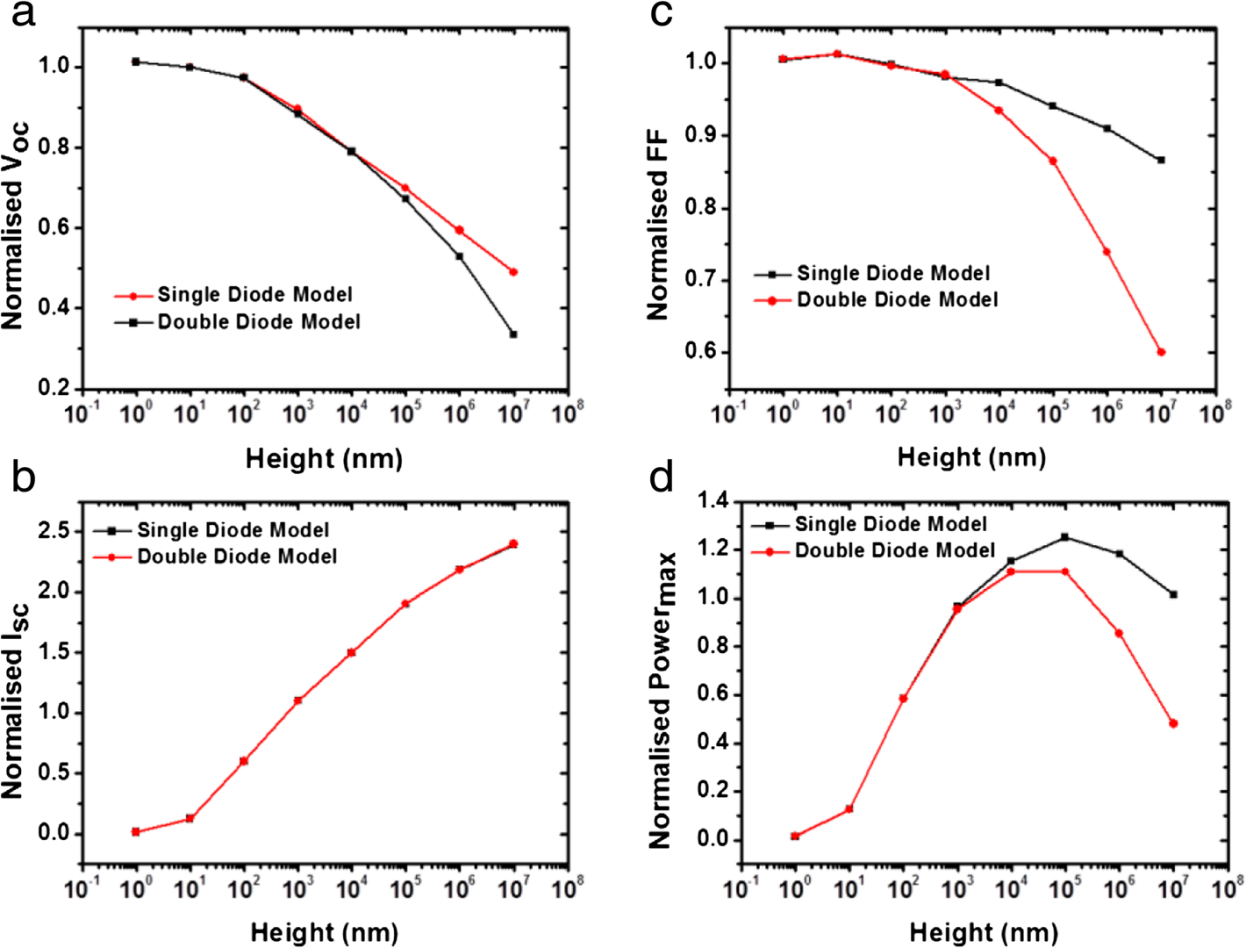
Simulated variation in the **a** open circuit voltage, **b** short circuit current, **c** fill factor, and **d** maximum power output with the orthogonal solar cell pillar height, normalized with respect to a planar solar cell. A reduction in the V_OC_ and FF and increase in the I_SC_, with pillar height increase, lead to an optimum pillar height for the highest efficiency gain. However, this gain is diminished for solar cells with a dominant recombination effect where the variation in device characteristics with illumination is the largest. These points to the critical role that recombination carrier losses play in orthogonal solar cells. This suggests that the orthogonal solar cells are better suited for devices with minimal recombination losses

Figure 4 shows the effect pillar height increase on the characteristics orthogonal solar cell relative to a planar solar cell. The results are normalized with respect to conventional planar device with a 300-nm-thick absorber layer and a back reflector. Figure 4a shows the degradation in the V_OC_ as the results of the non-uniform illumination across the various segments of the orthogonal device. This degradation is more pronounced when accounting for recombination effect. As expected, the longer pillars lead to the enhanced light absorption, and therefore an increase in the I_SC_, as shown in the Fig. 4b. An increase of the pillar height strongly affects the FF when considering the recombination effect, as depicted in Fig. 4c. This is expected as carrier losses become more significant at lower illumination intensities. As highlighted in the Fig. 4d, the combined effects of reduction in the V_OC_ and FF, and increase in the I_SC_ with an increase in the pillar height, lead to an optimum height for maximum solar cell efficiency. The optimum height depends on the device characteristics. In the ideal case where the carrier recombination in the photoabsorber layer is neglected, the solar cell efficiency improved by a factor of 1.2. However, the recombination severely degrades any gain from the efficiency from the use of orthogonal devices due to the significant drop in the fill factor. Surface recombination in p-i-n type structures considered in this work is likely to be minimal. This suggests that the orthogonal solar cells are better suited for devices with minimal recombination losses and exhibit minimal change in the V_OC_ and FF with the change in the illumination intensity. These results are consistent with the works of BM Kayes et al. [3] who report a peak in the orthogonal cell efficiency particularly at higher trap density. The key difference here is that in this work, orthogonal PV cells based on thin film materials are considered, whereas earlier works such as BM Kayes et al. consider orthogonal devices based on bulk semiconductors with low defect densities. The high defect density in thin film materials limits the interaction through depletion layer between adjacent circuit elements in the model used. This further enhances the effect of non-uniform illumination. For example, BM Kayes et al. report a constant fill factor with the variation pillar height for orthogonal devices based on bulk semiconductors. However, as shown in Fig. 4c, in this work, we report a reduction in the fill factor with increase pillar height in the case of orthogonal devices based on thin film semiconductors.

### Electric Field Confinement

**Fig. 5:**
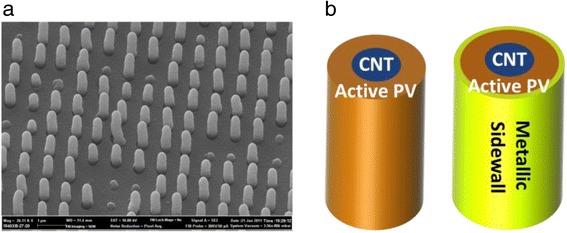
**a** SEM image of solar cell with orthogonal pillars consisting of vertical array of CNT coated with a-Si:H solar cell. The size effect of the CNT leads to an electric field enhancement within the device. **b** Illustrates two different approaches for the device fabrication, namely coating the outer wall with a metallic film or relying on the doped semiconductor as a section of the electrode results in a differences in the electric field strength within the device

Electric field enhancement effect in nanostructures such as carbon nanotubes (CNT) and metallic nanowires has been widely reported [6, 23–25]. Nanoscale dimensions in such structures typically result in the confinement of the electric field to a small area and subsequent increase in the electric field compared to a planar structure. The electric field enhancement has been exploited in the devices such as photodiodes and field emitters. Typically, orthogonal solar cells incorporate pillars with similar features within the device structure, thus making the electric filed confinement in orthogonal solar cells a potentially significant effect to consider. Figure 5a shows an array of orthogonal solar cells consisting of vertical conductive electrodes at the center of pillars, CNT in here, coated with thin film amorphous silicon p-i-n solar cell. The full device fabrication process and characteristics has been reported earlier [6]. As illustrated in the cross-sectional diagram in Fig. 5b, the final sidewall may consist of a metallic film, or left as the doped layer of the solar cell. The selection of the outer sidewall configuration would have an implication on the device characteristics.

The electric field penetration depth of metals is significantly shorter than the thickness of the metallic film on the outer shell. Therefore, the metallic sidewalls can be considered as the boundary point when calculating the electric field within the device. Based on this electric field, *E*, the function of the location along the pillar radius *x* can be calculated as shown in Eq. (6). Here, *r*_2_ and *r*_1_ are the outer and inner radiuses of the conductive metallic electrodes, respectively.

6 $$ E(x)=\frac{V}{x\; \ln \left[\frac{r_2}{r_1}\right]} $$

As evident in Eq. (6), electric field has maximum value of $\frac{V}{r_1\; \ln \left[\frac{r_2}{r_1}\right]}$ close to the central nanopillar, and the minimum value of $\frac{V}{r_2\; \ln \left[\frac{r_2}{r_1}\right]}$ at the outer sidewall metallic electrode. This variation in the electric field can be compared with the electric field in a planar structure consisting of parallel electrodes with a distance of *r*_2_–*r*_1_. Here, the ratio of the electric field at distance *x* from the center of the pillar long its radius, with respect to the electric field of a planar structure represented by factor *α*, can be defined as Eq. (7).

7 $$ \alpha =\frac{E(x)}{E_{\mathrm{planar}}}=\frac{r_2-{r}_1}{x\; \ln\ \left[\frac{r_2}{r_1}\right]} $$

In the case of device shown in the Fig. 5a with inner and outer radiuses of 50 and 350 nm, respectively, the structure with a metallic sidewall coating would exhibit an electric field enhancement factor of 3.1 close to the central pillar and a reduction in the electric field by a factor of 0.44 times close to the outer sidewall, relative to planar structure. Consider a p-i-n junction a-Si:H solar cell, which is a discrete heterojunction thin film solar cell dominated by drift process. Electric field across such device is a significant factor in the collection efficiency of photogenerated carriers. In such device, typically any gain due to electric field enhancement close to the pillar electrode is quenched by the reduction in the electric field close to the outershell electrode. The devices overall performance will be limited by the collection efficiency of the carriers close to the outer sidewall. A different outcome can be envisaged in the case of devices dominated by diffusion and dissociation processes, such as bulk heterojunction organic solar cells or single-crystal silicon solar cells. Although the electric field does not play a role in the separation of the photogenerated carries, it nevertheless affects the performance of the device through influencing the contact resistance and charge transfer to the electrodes.

Given the pillar lengths of up to few micrometers, it is feasible to omit the outer metallic sidewall and use a highly doped semiconductor layer on side walls in conjunction with a metallic mesh film at the base of the pillar as charge collection layer. In this configuration, the charge generated in the intrinsic layers are collected through the CNT and the n-type doped layer and transferred onto the bottom of the pillar where metal contacts provide a low impedance path for the collection of the carriers. The difference between the electric field penetration depth in doped semiconductor film compared with that of metal film leads to different electric field distribution within the structure. The electric field penetration depth of metallic films is in the order of few angstroms [26] and therefore few orders of magnitude thinner than thickness of the metal electrodes used in devices, whereas electric field penetration depth is comparable to a typical doped layer in the range of nanometers [27]. Metal outer shell of nanopillar solar cell acts as the boundary for the electric field, while without the metal outer shell, the electric field extends into the final dope layer, effectively increasing in the *r*_2_ in Eq. (7), and thus mitigates the quenching effect of outer sidewall. The electric field enhancement can be advantageous for the performance of certain type of devices. It has been shown to improve the charge collection efficiency in the organic solar cells [23, 28, 29] and enhance the operating speed and response of photodiodes [6].

### Series Resistance

**Fig. 6:**
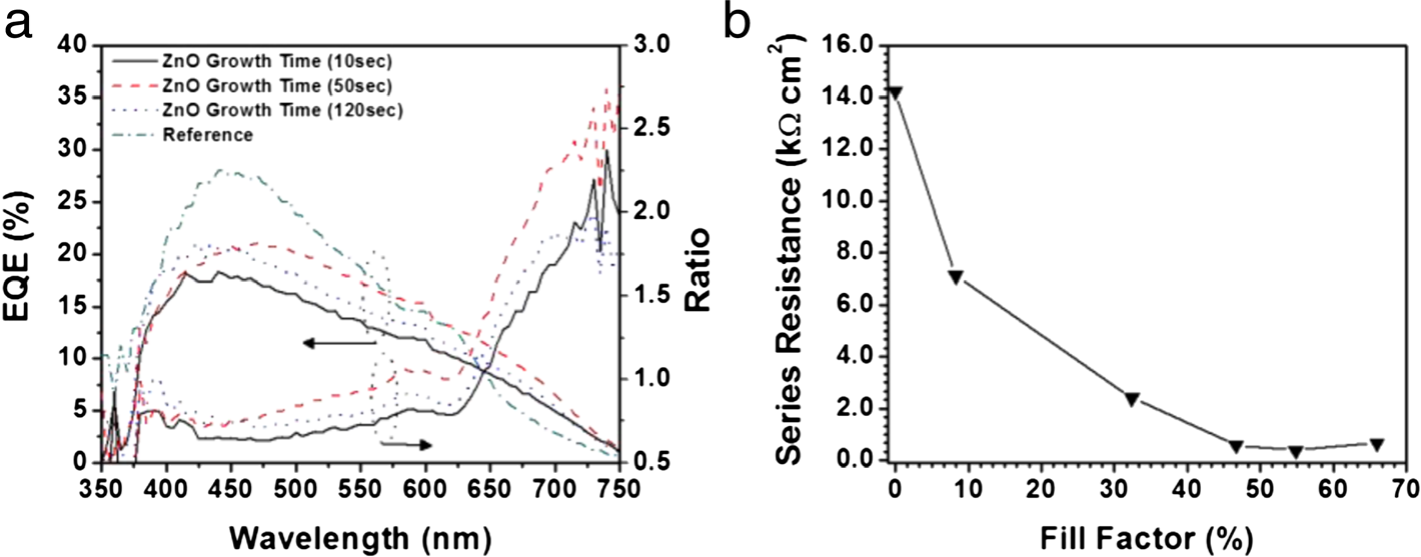
**a** Variation of EQE of a-Si:H thin film solar cell with growth time of ZnO nanorods. The long growth leads to a denser and larger nanorods, providing a longer optical path, which in turn enhances the collection of efficiency at longer wavelengths. This is demonstrated by up to ~2.5 times increase in the EQE at 750 nm. The rise in the EQE is accompanied with reduction in the solar cell’s FF. This reduction can be attributed to increase in the series resistance due to the increase in density and size of ZnO nanorods. **b** Variation in the FF of the cells with series resistance extracted from the I–V characteristics of PV device. The series resistance corresponds to the overall device series resistance. Each point in the graph relates to a different ZnO growth time. Here, longer ZnO growth time results in increased series resistance and reduced fill factor

Series resistance plays a critical role in the performance of photovoltaic devices. The large geometric surface area of orthogonal solar cells compared with planar ones makes these devices more susceptible to adverse impacts of the series resistance. Effects such as non-uniformity in the thickness and morphology of the sidewall electrodes and nanorode scaffold material properties can significantly influence the performance of orthogonal devices through their contribution to series resistance of the device. The effect of series resistance on orthogonal solar cell’s performance is illustrated in Fig. 6a. Here, the growth time of ZnO nanorods, which are used as scaffolds, on the external quantum efficiency (EQE) of orthogonal solar cells is shown. The growth time corresponds to the density and size of the ZnO nanorods, which in turn controls the geometric surface area of the device. The fabrication method and full device characterization has been present in earlier [30, 31]. Despite the excellent optical properties of ZnO nanorods [32], they exhibit low conductivities in the order of 0.001 S.m. As evident from the EQE spectrum in Fig. 6a, longer and denser ZnO nanorods, with the longest growth time, improve the response of the solar cell at longer wavelength, where the gain from orthogonal cells is greatest. However, despite this gain, the overall performance of the cell is reduced, due to the effect of series resistance introduced by the ZnO nanorod scaffold as illustrated in Fig. 6b. Here, the reduction in the FF with the increase in the ZnO growth time, and subsequent increased in geometric surface area, can be clearly attributed to the series resistance of the device. Each point of the graph represents an orthogonal solar cell grown using different ZnO growth duration.

**Fig. 7:**
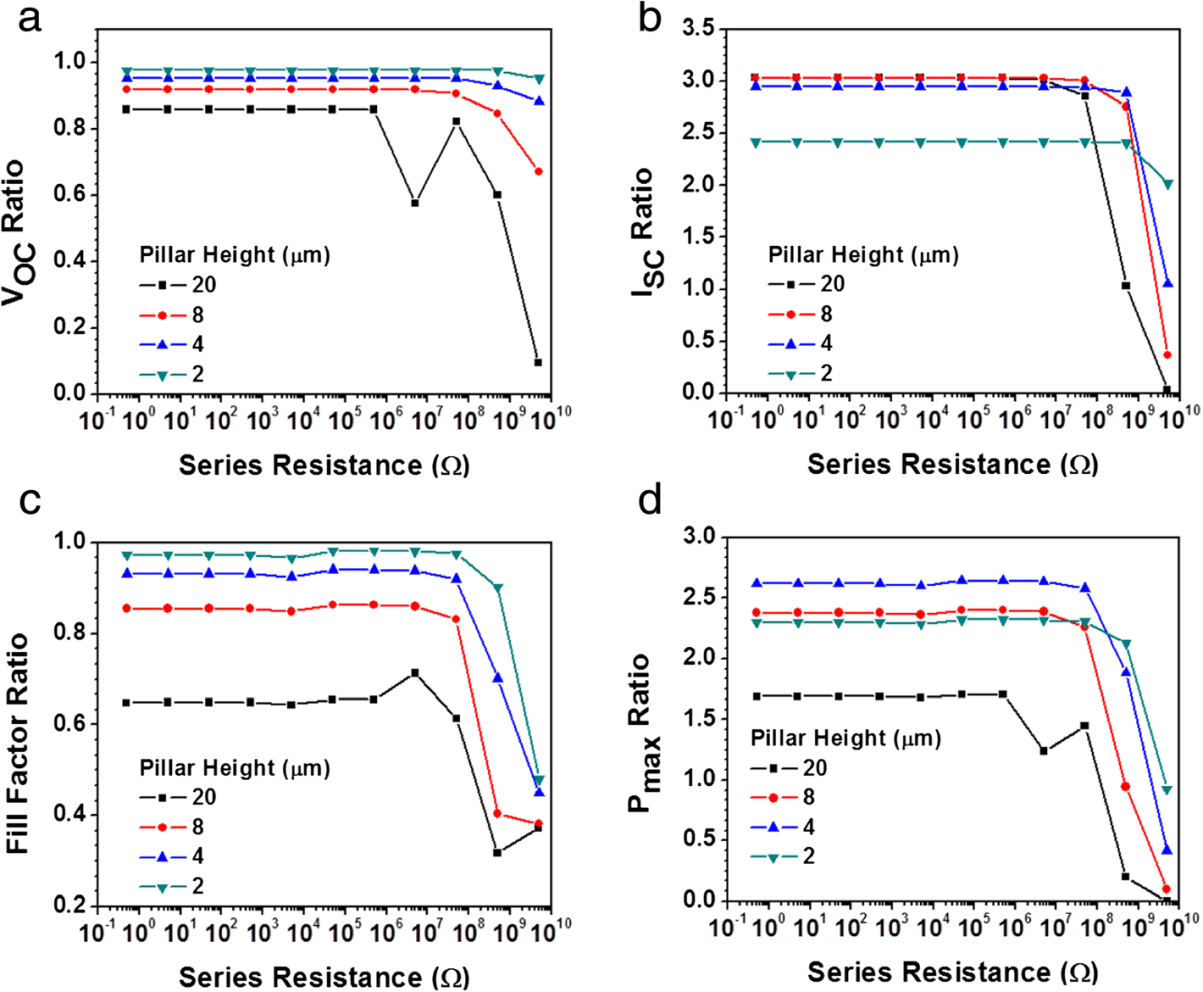
Simulated variation in **a** open circuit voltage, **b** short circuit current, **c** fill factor, and **d** maximum output power with series resistance of each segment of orthogonal solar cell. Pillar height is represented by the number of segments. Series resistance strongly affects the performance of the device from around 107 Ω, leading to a significant degradation in the device performance, particularly for longer pillar heights

The influence of the series resistance in orthogonal solar cells can be examined theoretically by modeling the device as a series of parallel-connected segments, as discussed in Fig. 2. It should be noted that when considering the series resistance, the segments furthest away from the measurement point experience the highest total series resistance due to the contribution of the series resistances of preceding segments. Figure 7 illustrates the simulated effect of series resistances on the performance of orthogonal devices with various pillar lengths measured at the base of the pillar. It can be seen that the response of various performance indicators are constants with the increase in segment series resistance up to the range of ~10^6^ Ω, after which a significant degradation can be observed. It should be noted that although pillar lengths of up to 20 μm is considered in here, these dimensions may only be feasible with careful consideration of aspect ratios and pitch and by using highly optimized film deposition/growth.

As shown in Fig. 7a, there is a significant drop in the V_OC_ with the increase in series resistance. This reduction is most pronounced for longer pillar. Indeed, the shortest pillar’s V_OC_ response is consistent with the response of planar devices where the V_OC_ is not expected to vary significantly with the increase in series resistance. The variation in the I_SC_ with the series resistance is more pronounced for the longer pillar as depicted in Fig. 7b. Here, high series resistance effectively decouples segments furthest away from the probing point and subsequently results in a sharp drop in the I_SC_ with increase in the series resistance, particularly in taller structures. Once again, this variation is minimal for orthogonal devices with shortest pillar highest, in line with the characteristics of planar devices. As shown in Fig. 7c, series resistance adversely affects the FF of the all orthogonal devices including shorter structures. The combination of these performance indices leads to the variation in the maximum power output with series resistance shown in Fig. 7d. It highlights the fact that the reduction in the P_Max_ and subsequently solar cell efficiency with increase in the series resistance is most pronounced in the case of taller structures. Reduction in pillar height reduces the influences of series resistance on the P_Max_ and the subsequent device efficiency.

For comparison, Table 1 shows the segment series resistance of various thin film materials calculated based on a 40-nm-long hallow cylindrical segment with an inner and outer radiuses of 300 and 320 nm, respectively. The resistivity of many of the conventional electrode materials, including doped semiconductors, is low enough to prevent any degradation in the performance of the orthogonal solar cells with pillar heights of 20 μm or less. However, the use of intrinsic films fails to achieve this for the case considered in this work.

**Table 1:** Resistivity and segment resistance of a number of different thin film materials used as the charge collection and scaffolding in vertical solar cells

Material	*σ* (Ω.m)	*R* (Ω)
Gold	2E-08	2E-02
Aluminum	2E-08	3E-02
ITO	1E-06	1
PEDOT:PSS	1E-05	10
n-type μc-Si	1E-04	100
n-type a-Si:H	0.1	1E + 05
p-type a-Si:H	10	1E + 07
Intrinsic silicon	640	8E + 08
Intrinsic ZnO	1000	1E + 09

Segment resistance is calculated based on a 40-nm-long hollow cylinder with outer and inner diameters of 320 and 300 nm, respectively

## Conclusions

In this work, the effects that influence the performance of orthogonal solar cells are investigated. It is shown that although orthogonal devices have a potential efficiency gain compared to planar devices, a careful design is required in order to achieve their optimum performance efficiency. We discuss the role of optical non-uniformity across the depth of the solar cell. As light travels through the orthogonal solar cell, its intensity and spectrum change. This change in the illumination translates in a variable output characteristics from parallel-connected segments of the solar cell. Lower segments, owing to their lower open circuit voltage due to lower intensity illumination, act as power sink consuming the power generated by the upper segments. This effect is worsened when considering the recombination losses in the device, which are prevalent in thin film solar cells.

Ordered array of nanopillars has widely been demonstrated as a means of enhancing broadband light absorption of materials. Device simulation suggests that in the case of closely packed nanopillar array, the light intensity decays cross the depth of the device [33]. However, in addition to the absorption, scattering plays an important role in the optical properties of nanopillar arrays [22]. A simple optical scattering may result in a more uniform illumination as a function of depth of the pillars. Despite this, the overall non-uniform illumination and the role it plays on the performance of orthogonal solar cells remain an issue to consider. The electric field enhancement due to the nanoscale size shape of the pillars used in orthogonal solar cells is discussed. The design of the pillar structure, in particular the effect of metallic side wall, on the electric field distribution of the device is considered and shown that with a careful design, it is possible to exploit this effect. It is shown that series resistance due to the additional surface structure created through the use of pillars can play an import role in the device performance. All of these effects have the potential for reducing the performance of orthogonal solar cells and should be considered in device designs.

This work has considered the influence of physical effects such as illumination non-uniformity, electric-field confinement, and series resistance separately in order to qualitatively examine the specific contribution of each of these on the operation of orthogonal PV devices. However, the interplay between these physical mechanism means coupled optical/electrical models [34–36] provide a more accurate quantitative description of the device performance.
